# Case report of gastric syphilis in Korea: Clinical features, pathology, management, and prognosis

**DOI:** 10.1097/MD.0000000000028212

**Published:** 2021-12-17

**Authors:** Hyung-Joo Yu, Seong-Jung Kim, Hyung-Hoon Oh, Chan-Mook Im, Bora Han, Eun Myung, Sook-Jung Yun, Kyung-Hwa Lee, Young-Eun Joo

**Affiliations:** a Department of Internal Medicine, Chonnam National University Medical School, Gwangju, Republic of Korea; b Department of Internal Medicine, Chosun University College of Medicine, Gwangju, Republic of Korea.; c Department of Dermatology, Chonnam National University Medical School, Gwangju, Republic of Korea; d Department of Pathology, Chonnam National University Medical School, Gwangju, Republic of Korea.

**Keywords:** stomach, syphilis

## Abstract

**Rationale:**

Syphilis is a contagious infectious disease caused by *Treponema pallidum*. Gastric involvement of syphilis is rare and has nonspecific gastrointestinal symptoms and endoscopic findings. To date, 16 cases have been reported in Korea. Here, we report 2 additional cases of gastric syphilis in men in their 30 second.

**Patients concerns:**

Two 35- and 33-year-old men presented with epigastric pain.

**Diagnosis:**

The serum venereal disease research laboratory and fluorescent treponemal antibody absorption tests were positive. Esophagogastroduodenoscopy showed multiple variable-sized flat elevated lesions and geographic ulcers with whitish exudates in the antrum and body. Warthin–Starry silver staining of endoscopic biopsy specimens confirmed gastric syphilis.

**Interventions:**

The patients were treated with an intramuscular injection of 2.4 million units of benzathine penicillin once a week for 3 weeks.

**Outcomes:**

Clinical symptoms and gastric lesions were completely resolved.

**Lessons:**

First, gastric syphilis, despite its rarity and nonspecific symptoms and endoscopic findings, should be considered in a rare extracutaneous presentation of syphilis. Second, a high index of clinical suspicion and an accurate diagnosis based on a combination of clinical, radiological, endoscopic, serologic, and histopathologic findings provide an opportunity to identify and treat patients with gastric syphilis.

## 1 Introduction

Syphilis is a contagious infectious disease caused by *Treponema pallidum*. Gastric involvement of syphilis is a rare extracutaneous presentation, occurring in less than 1% of patients with syphilis. It is difficult to be diagnosed because of the lack of pathognomonic clinical, radiologic, endoscopic, and histopathologic findings.^[1–5]^ Therefore, the diagnosis of gastric syphilis is usually based on a combination of clinical, radiological, endoscopic, serologic, and histopathologic findings. To date, 16 cases have been reported in Korea.^[6–15]^ Here, we report 2 cases of gastric syphilis as a rare extracutaneous presentation of syphilis and review the literature pertaining to this condition.

## 2 Case reports

### 2.1 Case 1

**Figure 1:**
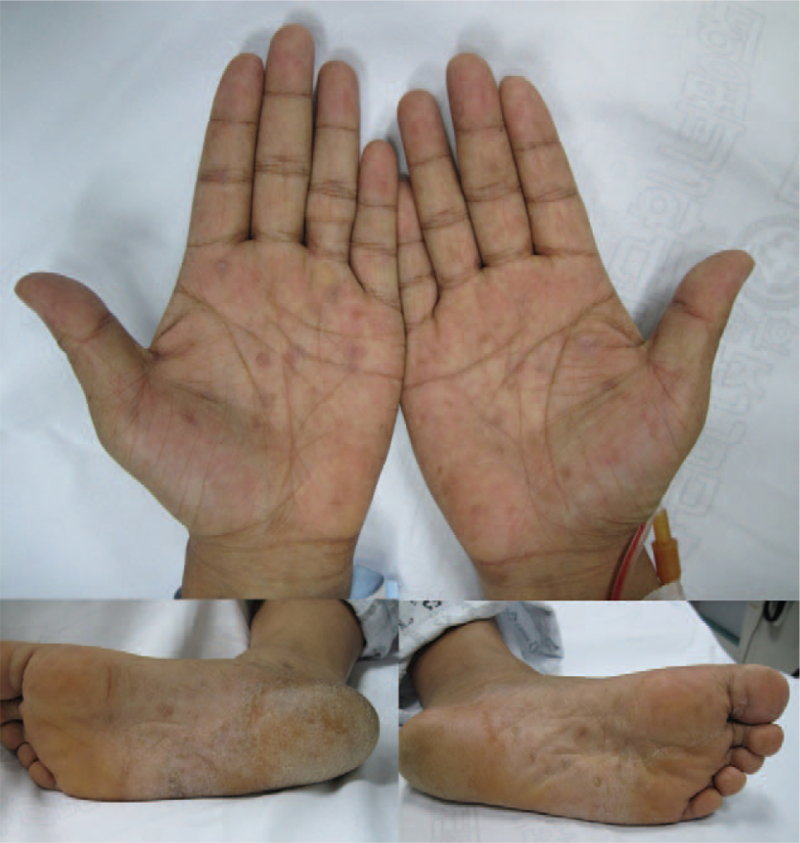
Skin appearance. Asymptomatic brownish variable-sized round-shaped macules with scales are seen on both palms and soles, which is consistent with secondary syphilis.

**Figure 2:**
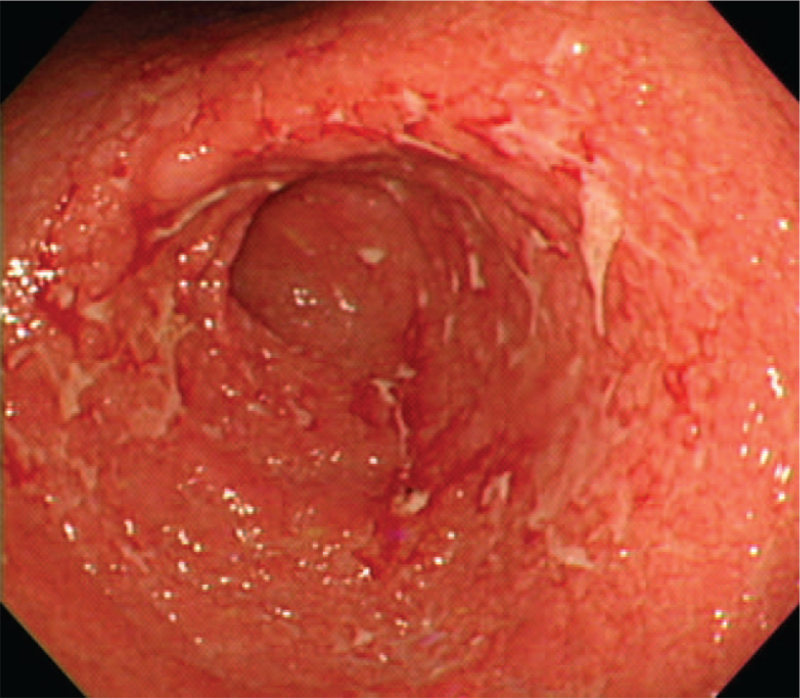
Endoscopic findings. Multiple geographic ulcers with covered whitish exudates in the antrum are seen.

**Figure 3:**
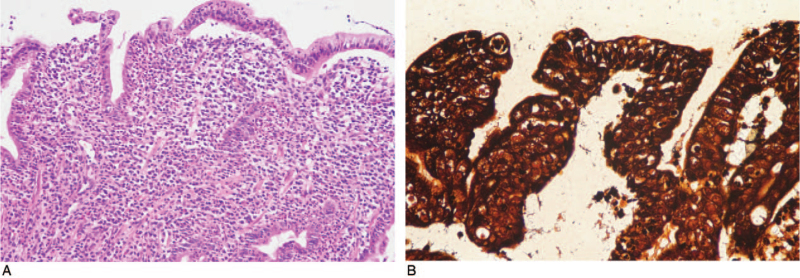
Microscopic findings. (A) A dense mononuclear cell infiltrate with prominent plasma cells in the lamina propria is observed (hematoxylin–eosin, × 200). (B) Numerous spirochetes are seen within the lamina propria (Warthin–Starry silver stain, × 400).

A 35-year-old man was admitted to our hospital with a 1-month history of epigastric pain, nausea, and vomiting. On admission, his vital signs were normal. He denied any previous medical history, including gastrointestinal disease, abdominal surgery, or significant medical illness. In addition, he was not taking any medications, including NSAIDs. Physical examination revealed asymptomatic brownish variable-sized round macules with scales on both palms and soles that had been present for several months (Fig. 1). Skin lesions were consistent with secondary syphilis. No oropharyngeal or genital lesions were observed. Mild abdominal tenderness was elicited in the epigastrium. He reported sexual intercourse with multiple sexual partners several months prior to admission. Laboratory evaluation revealed normal hemoglobin, hematocrit, white blood cell count, and hepatic and renal function. The C-reactive protein level was elevated at 2.96 (normal, 0–0.3) mg/dL. The serum venereal disease research laboratory test was positive, with a titer of 1:8, and the fluorescent treponemal antibody absorption (FTA-ABS) test was reactive. Serum human immunodeficiency virus (HIV) antibodies were negative. Esophagogastroduodenoscopy (EGD) revealed multiple geographic ulcers with easy touch bleeding and whitish exudates in the antrum (Fig. 2). The esophagus, body and fundus of the stomach, and duodenum were normal. Endoscopic biopsies taken from the antrum showed a dense mononuclear cell infiltrate with prominent plasma cells on hematoxylin–eosin staining (Fig. 3A). *Helicobacter pylori* was not detected by Giemsa staining. Because syphilis was suspected based on the results of skin lesions and serologic tests, Warthin–Starry silver staining was subsequently performed, and numerous spirochetes were identified in the lamina propria, confirming gastric syphilis (Fig. 3B). He was treated with an intramuscular injection of 2.4 million units of benzathine penicillin once a week for 3 weeks. His clinical symptoms resolved within 5 days of treatment initiation. Follow-up EGD after 2 months showed complete resolution of multiple geographic ulcers. Biopsies of the gastric mucosa were negative for spirochetes, and the lymphocytic infiltrate resolved. Informed consent was obtained from the patient for the purpose of publication.

### 2.2 Case 2

**Figure 4:**
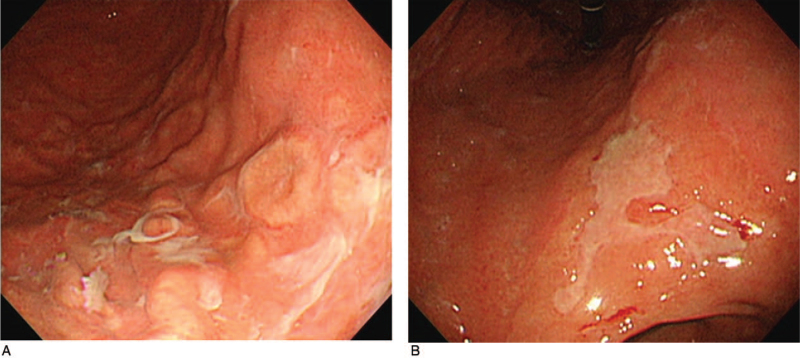
Endoscopic findings. (A) Multiple variable-sized whitish discolored flat elevated lesions with focal erosion and ulceration on the body. (B) Large geographic ulcers with regular edges and even whitish exudates on the lesser curvature side of the lower body.

A 33-year-old man presented at the outpatient clinic with a 1-month history of epigastric pain. Physical examination revealed mild abdominal tenderness in the epigastrium. Laboratory evaluation revealed normal hemoglobin, hematocrit, white blood cell count, and hepatic and renal function. The serum treponema pallidum hemagglutination test was positive, with a titer of 1:1280, and the FTA-ABS test was reactive. Serum HIV antibodies were negative. EGD showed multiple variable-sized whitish discolored flat elevated lesion with focal erosion and ulceration on the body (Fig. 4A) and large geographic ulcers with regular edges and even whitish exudates on the lesser curvature side of the lower body (Fig. 4B). Warthin–Starry silver staining of endoscopic biopsy specimens confirmed gastric syphilis. He was treated with an intramuscular injection of 2.4 million units of benzathine penicillin once a week for 3 weeks. The patient's clinical symptoms and gastric lesions were completely resolved. Informed consent was obtained from the patient for the purpose of publication.

## 3 Discussion

Syphilis is a chronic systemic infection caused by *Treponema pallidum*, which is mostly transmitted via sexual contact and blood. Gastric involvement is a rare presentation, observed in less than 1% of patients.^[1–5]^ To date, 18 cases of gastric syphilis (including the present cases) have been reported in Korea (Table 1).^[6–16]^ The patients with gastric syphilis were aged 20–57 (mean, 31.7) years and consisted of 10 men and 8 women. The most common symptoms were epigastric pain and discomfort (88.9%, 16/18 cases), followed by nausea, vomiting, indigestion, anorexia, melena, and weight loss. These clinical findings are nonspecific manifestations and are similar to those reported in the English literature.^[1–5]^

**Table 1:** Summary of reported cases of gastric syphilis in Korea.

Patient No.	Author, year	Age (years old)/sex	Symptoms (duration)	Endoscopic description	Location	VDRL/TPHA/FTA-ABS/HIV	Detection of *Treponema pallidum*	Cutaneous manifestations	Other extracutaneous manifestations	Treatment	Symptom and endoscopic improvement	Follow-up
1	Kim et al, 1981^[6]^	33/female	Epigastric pain and discomfort, anorexia, weight loss (2 months)	Multiple irregular shaped erosions	Antrum, body	1:8/+/NA/NA	NA	NA	Liver, neurosyphilis	Benzathine penicillin G IM	4–5d/2 mo	2 mo
2	Kim et al, 1981^[6]^	24/female	Vomiting, epigastric discomfort (1 month)	Multiple irregular shaped ulcers	Antrum, body	+/+/NA/NA	NA	NA	NA	Benzathine penicillin G IM	NA/3 mo	3 mo
3	Kim et al, 1981^[6]^	20/female	Indigestion, epigastric pain, vomiting (3 mo)	Erosion	Antrum, body	1:32/+/NA/NA	NA	NA	NA	Benzathine penicillin G IM	25 d/NA	25 d
4	Kim et al, 1981^[6]^	23/female	Epigastric pain, vomiting (20 d)	Multiple irregular shaped ulcers and erosions, hypertrophy of rugae	Antrum, body	+/+/NA/NA	NA	NA	NA	NA	NA	NA
5	Kim et al, 1981^[6]^	35/female	Indigestion, epigastric pain, vomiting (3 months)	Multiple small ulcers, mucosal hypertrophy	Antrum, body	+/NA/NA/NA	NA	NA	NA	Benzathine penicillin G IM	NA	NA
6	Chung et al, 1989^[7]^	21/female	Epigastric pain, vomiting (1 month)	Multiple erosive hemorrhagic patchs	NA	1:64/1:1280//+/NA	Fluorescent treponemal antibody absorption complement staining (+)	Palm, sole	NA	Benzathine penicillin G IM	NA/NA	NA
7	Kwon et al, 2004^[8]^	43/male	Indigestion, epigastric discomfort, Skin eruption, both lower extremity pitting edema (2 weeks)	Multiple shallow ulcers with varying degrees of nodular mucosa	Antrum	-/-/+/NA	NA	Genital area, trunk, palm, sole	Liver, kidney	Benzathine penicillin G IM	16 d/1 mo	8 wks
8	Lee et al, 2006^[9]^	46/male	Nausea, vomiting, melena (6 days)	Huge ulcerative lesion with diffuse irregular and nodular mucosa	Antrum	1:32/1:160/NA/NA	Warthin-Starry silver staining (+)	Genital area (2 y ago)	Negative	Benzathine penicillin G IM	3 d/NA	NA
9	Ji et al, 2006^[10]^	21/female	Epigastric pain, nausea, vomiting (2 months)	Uneven coarse edematous mucosa, shallow ulceration	Antrum, body	+/+/NA/NA	NA	Genital area	NA	Benzathine penicillin G IM	NA/2 months	2 months
10	Choi et al, 2006^[11]^	43/male	Epigastric tenderness, anorexia (1 month)	Diffuse mucosal nodularity with several ulcerations with interveneing hyperemic friability.	Antrum, body	1:64/NA/+/-	Warthin-Starry silver staining (+)	Negative	Negative	Benzathine penicillin G IM	NA	N/A
11	Park et al, 2008^[12]^	25/male	Epigastric pain, vomiting (1 mo)	Geographic irregular ulcer and shallow depressed mucosal lesions	Antrum	NA/+/NA/NA	Warthin-Starry silver staining (+)	NA	Duodenum	Benzathine penicillin G IM	NA	NA
12	Jeong et al, 2008^[13]^	57/male	Epigastric pain, indigestion, nausea, headache (3 mo)	Multiple nodular mucosal changes and multiple variable sized depressed lesions	Antrum, body	1:512/1:320/NA/-	Warthin-Starry silver staining (-)	Negative	Neurosyphilis	Benzathine penicillin G IM	7 d/3 mo	6 mo
13	Kim et al, 2009^[14]^	25/male	Epigastric pain (3 wks)	Multiple round whitish patches of variable size, irregular shaped shallow ulcers and erosions with easy bleeding	Antrum	+/NA/+/-	Warthin-Starry silver staining (+)	NA	NA	Benzathine penicillin G IM	NA/2 mo	2 mo
14	Kim et al, 2009^[14]^	32/male	Epigastric pain, general weakness, indigestion, abdominal discomfort, melena (10 d)	Numerous erosive or ulcerative lesions with whitish border	Antrum, body	+/NA/+/-	Warthin-Starry silver staining (+)	Genital area	NA	Benzathine penicillin G IM	NA/36 d	36 d
15	Kim et al, 2012^[15]^	21/female	Nausea, vomiting, weight loss (2 mo)	Hypertrophied gastric rugal folds with several ulcerations	Antrum, body	+/NA/+/NA	NA	NA	NA	Benzathine penicillin G IM	NA	NA
16	Roh et al, 2015^[16]^	34/male	Epigastric pain, febrile sense, generalized edema, inguinal rash (3 wks)	Multiple irregular shallow ulcers with whitish exudates and central depression	Antrum, body, fundus, cardia	1;256/+/+/-	NA	NA	Kidney	Benzathine penicillin G IM	NA	NA
17	Present case	35/male	Epigastric pain, nausea, vomiting (1 mo)	Multiple geographic ulcers with easy touch bleeding and whitish exudates	Antrum	1;8/NA/+/-	Warthin-Starry silver staining (+)	Palm, sole	NA	Benzathine penicillin G IM	5 days/2mo	2 mo
18	Present case	33/male	Epigastric pain, nausea, vomiting (1 mo)	Multiple variable sized whitish discolourised flat elevated lesion with focally erosions and ulcerations, large geographic ulcer with regular edge and even whitish exudate base	Body	NA/1;1280/+/-	Warthin-Starry silver staining (+)	Genital area (2 mo ago)	NA	Benzathine penicillin G IM	NA	NA

FTA-ABS = fluorescent treponemal antibody absorption, HIV = human immunodeficiency virus, IM = intramuscular injection, NA = not available, TPHA = treponema pallidum hemagglutination, VDRL = venereal disease research laboratory.

In a review of the English literature, approximately 13% had a history of syphilis diagnosis, and 33% had concurrent clinical manifestations of syphilis, including genital ulcer, rash, and lymphadenopathy.^[1]^ In our study, 2 cases (11.1%) with gastric syphilis had a previous history of syphilis diagnosis, and5 cases (27.8%) had concurrent clinical cutaneous manifestations of syphilis. Of the concurrent clinical cutaneous manifestations, the genital area was involved in 3 cases, and the palm and sole were involved in 3 cases, including our case.

Syphilis is a systemic disease that can spread to various organ systems, including the liver, gastrointestinal tract, kidneys, structures of the eye, and neurovascular system.^[1–5]^ In our study, 4 cases (22.2%) had other extracutaneous manifestations, including 2 cases each in the liver and the kidney, and 2 cases of neurosyphilis.

In serology data, the nontreponemal test or treponemal tests (treponema pallidum hemagglutination or FTA-ABS) were available for all cases reviewed except 2 cases or one case, respectively. The nontreponemal test was positive in 15 out of 16 cases. The treponemal tests were positive in all 17 cases. Serum HIV testing was conducted in 7 cases, all of which were negative.

With regard to endoscopic features, the antrum and body were the most frequently involved, and multiple erosion, ulceration, nodularity, and mucosal fold hypertrophy were predominantly observed. As such, endoscopic findings of patients with gastric syphilis vary and should be differentiated from advanced gastric cancer, lymphoma, tuberculosis, Crohn's disease, sarcoidosis, and eosinophilic gastritis. In particular, in some cases, it may not be easy to distinguish malignant diseases such as advanced gastric cancer and lymphoma; therefore, primary diagnosis of gastric syphilis is very difficult. However, if diagnosis is delayed, it can be treated surgically either because of complications such as gastric perforation and outlet obstruction or because of strong suspicion of advanced gastric cancer or lymphoma.^[1–5]^ In both cases of our study, clinical, radiologic, and endoscopic findings indicated linitis plastica. However, it could not be diagnosed via an endoscopic biopsy. In both cases, gastric syphilis was diagnosed via endoscopic mucosal resection and exploratory laparotomy.^[10,11]^

Routine hematoxylin and eosin staining of endoscopic biopsy specimens of gastric lesions typically shows chronic inflammation with dense plasma cell infiltration in the lamina propria. Histopathologic diagnosis of gastric syphilis can be made by identifying *Treponema pallidum* in gastric lesions via Warthin–Starry silver staining, immunofluorescence, or polymerase chain reaction.^[17–19]^ In our study, *Treponema pallidum* was detected in biopsied specimens via Warthin–Starry silver staining and FTA-ABS complement staining in 8 cases and 1 case, respectively, and was negative in only one of the Warthin–Starry silver staining cases.

An intramuscular injection of benzathine penicillin G is recommended as an appropriate antibiotic for the treatment of gastric syphilis. In principle, the duration of treatment should be determined according to the stage of syphilis.^[1–5]^ In our study, the majority of patients with gastric syphilis were treated with an intramuscular injection of 2.4 million units of benzathine penicillin once a week for 3 weeks.

Appropriate antibiotic treatment of gastric syphilis improves clinical symptoms and gastric lesions.^[1–5]^ In a previous report, clinical symptoms usually disappeared in approximately 3 to 4 days, and gastric lesions began to improve as early as 10 days after the initiation of treatment.^[2]^ In our study, clinical symptoms improved after 3 to 25 days, and endoscopic findings usually improved after 1 to 3 months of treatment.

In summary, first, gastric syphilis, despite its rarity and nonspecific symptoms and endoscopic findings, should be considered in a rare extracutaneous presentation of syphilis. Second, a high index of clinical suspicion and an accurate diagnosis based on a combination of clinical, radiological, endoscopic, serologic, and histopathologic findings provide an opportunity to identify and treat patients with gastric syphilis.

## Author contributions

**Conceptualization:** Young Eun Joo.

**Investigation:** Hyung-Joo Yu, Seong-Jung Kim.

**Resources:** Hyung-Hoon Oh, Chan-Mook Im, Bora Han, Eun Myung, Sook-Jung Yun, Kyung-Hwa Lee.

**Supervision:** Young Eun Joo.

**Writing – original draft:** Hyung-Joo Yu, Seong-Jung Kim, Young Eun Joo.

**Writing – review & editing:** Hyung-Hoon Oh.
